# Rectal HSV-2 Infection May Increase Rectal SIV Acquisition Even in the Context of SIVΔnef Vaccination

**DOI:** 10.1371/journal.pone.0149491

**Published:** 2016-02-17

**Authors:** Natalia Guerra-Pérez, Meropi Aravantinou, Filippo Veglia, Diana Goode, Rosaline Truong, Nina Derby, James Blanchard, Brooke Grasperge, Agegnehu Gettie, Melissa Robbiani, Elena Martinelli

**Affiliations:** 1 Center for Biomedical Research, Population Council, New York, New York, United States of America; 2 Tulane National Primate Research Center, Tulane University, Covington, Louisiana, United States of America; 3 Aaron Diamond AIDS Research Center, Rockefeller University, New York, New York, United States of America; University of Pittsburgh Center for Vaccine Research, UNITED STATES

## Abstract

Prevalent HSV-2 infection increases the risk of HIV acquisition both in men and women even in asymptomatic subjects. Understanding the impact of HSV-2 on the mucosal microenvironment may help to identify determinants of susceptibility to HIV. Vaginal HSV-2 infection increases the frequency of cells highly susceptible to HIV in the vaginal tissue of women and macaques and this correlates with increased susceptibility to vaginal SHIV infection in macaques. However, the effect of rectal HSV-2 infection on HIV acquisition remains understudied. We developed a model of rectal HSV-2 infection in macaques in combination with rectal SIVmac239Δnef (SIVΔnef) vaccination and our results suggest that rectal HSV-2 infection may increase the susceptibility of macaques to rectal SIVmac239 wild-type (wt) infection even in SIVΔnef-infected animals. Rectal SIVΔnef infection/vaccination protected 7 out of 7 SIVΔnef-infected macaques from SIVmac239wt rectal infection (vs 12 out of 16 SIVΔnef-negative macaques), while 1 out of 3 animals co-infected with SIVΔnef and HSV-2 acquired SIVmac239wt infection. HSV-2/SIVmac239wt co-infected animals had increased concentrations of inflammatory factors in their plasma and rectal fluids and a tendency toward higher acute SIVmac239wt plasma viral load. However, they had higher blood CD4 counts and reduced depletion of CCR5+ CD4+ T cells compared to SIVmac239wt-only infected animals. Thus, rectal HSV-2 infection generates a pro-inflammatory environment that may increase susceptibility to rectal SIV infection and may impact immunological and virological parameters during acute SIV infection. Studies with larger number of animals are needed to confirm these findings.

## Introduction

Prevalent Herpes Simplex Virus type 2 (HSV-2) infection is associated with a 2- to 4-fold increased risk of Human Immunodeficiency Virus (HIV) acquisition[1–3]. Although this can be partially explained by the presence of genital and rectal ulcers and inflammation at the site of HSV-2 infection in symptomatic subjects, the association remains in asymptomatic subjects in absence of detectable active HSV-2 replication [3–6]. Clarifying the mechanisms involved in the increased susceptibility of HSV-2 positive individuals to HIV infection may help to define the characteristics of mucosal microenvironment that facilitate HIV transmission. About 1 in 6 Americans between the ages of 14 and 49 are infected with HSV-2 and, although HSV-2 infection is more prevalent in women, about 11% of US men are infected with HSV-2 and HSV-2 prevalence reaches 40% in high-risk male populations in US and areas of Sub-Saharan Africa[7, 8]. Notably, two studies on HSV-2 infection among HIV negative men who have sex with men (MSM), found that HSV-2 shedding (subclinical and symptomatic) is predominantly perianal[9, 10] and although data are lacking, anorectal HSV-2 infection is likely a significant burden in the female population as well [11]. Nonetheless, the impact of rectal HSV-2 infection on rectal SIV acquisition is understudied.

Using a macaque model of vaginal HSV-2 infection, we have shown that HSV-2 infected asymptomatic macaques have an increased frequency of α_4_β_7_^high^ CD4^+^ T cells in their vaginal tissue and are more susceptible to vaginal SHIV infection[12]. This has been confirmed in HSV-2-infected asymptomatic women[5], in which an increased presence of CCR5^+^ HIV target cells at the site of HSV-2 infection has also been reported[13]. In a previous study, we used a model of HSV-2 rectal infection in macaques to show that HSV-2 shedding can be detected in rectal swabs at least during acute infection and in absence of lesions[14]. Using this model, we found that rectal HSV-2 infection increases the frequency of α_4_β_7_^high^ CD4^+^ T cells in the rectal tissue[14], which is associated with increased susceptibility to rectal SIVmac239wt infection[15]. In the present study, we explored how rectal HSV-2 infection would impact rectal SIVmac239 infection, also in the context of infection with a live-attenuated SIV vaccine (LAV).

LAVs effectively protect macaques against genetically related virus challenges[16–20] and, although they may never be used in humans because of safety concerns [21, 22], understanding the nature of their protection may be useful for the development of other vaccine approaches [23]. The degree of protection correlates inversely with the degree of attenuation of the LAV and with the level of sequence variation between the vaccine and infectious strains[24]. Intravenous (i.v.) vaccination with SIVmac239ΔNef (SIVΔNef) is virtually 100% effective against sequence-matched wild-type SIVmac239 (SIVmac239wt) challenge (i.v. and mucosal) and 95% effective against genetically related SIVmac251[23, 25, 26]. It was shown that progesterone treatment, which increases susceptibility to vaginal SIV infection[27], decreases the efficacy of vaccination with SHIV89.6[28]. We evaluated the effects of HSV-2 infection on the protection mediated by the prototypical LAV SIVΔNef.

We found that rectal HSV-2 infection induces a pro-inflammatory environment and our results suggest that rectal HSV-2 infection may increase susceptibility to rectal infection with SIVmac239wt and drives higher acute SIV plasma viral load (VL). Moreover, the data suggest that HSV-2 infection may undermine the protective effect mediated by SIVΔNef rectal infection/vaccination. Studies with larger number of animals are needed to confirm these findings.

## Materials and Methods

### Ethics Statement

Herpes B negative male Indian rhesus macaques (*Macaca mulatta*, mean age: 9 years range: 5–13 years; mean weight: 12.93 kg range: 8.0–17.8 kg), 32 animals in total, were housed at Tulane National Primate Research Center (TNPRC; Covington, LA). All animal care procedures were compliant under regulations of the Animal Welfare Act, and the Guide for the Care and Use of Laboratory Animals. All protocols and studies were reviewed and approved by the Institutional Animal Care and Use Committee (IACUC) of TNPRC (OLAW Assurance #A4499-01). TNPRC is accredited by the Association for Assessment and Accreditation of Laboratory Animal Care (AAALAC#000594). A team of veterinarians and technicians monitored the well-being of the animals and provided direct support to minimize stress during the study period. The macaques were socially housed, indoors and in climate controlled restrictions with a 12/12-light/dark cycle, justified and approved by the IACUC as part of the protocol review. All the animals on this study were monitored twice daily to ensure their welfare. Any abnormalities, including those of appetite, stool, behavior, were recorded and reported to a veterinarian. The animals were fed commercially prepared monkey chow twice daily. Water was available at all times through an automatic watering system. Supplemental foods were provided in the form of fruit, vegetables, and foraging treats as part of the TNPRC environmental enrichment program. The TNPRC environmental enrichment program is reviewed and approved by the IACUC semiannually. Animals were anesthetized with ketamine-HCl (10mg/kg) or tiletamine/zolazepam (6mg/kg), prior to blood collections and biopsies, and treated with buprenorphine (0.01mg/kg body weight) for analgesia. The monkeys were euthanized at the end of the study with tiletamine/zolazepam (8mg/kg body weight) and buprenorphine (0.01mg/kg body weight) followed by an overdose of pentobarbital sodium. None of the animals became severely ill or died prior to the experimental endpoint. The TNPRC policy for early euthanasia/humane endpoint was included in the protocol in case those circumstances arose.

### Macaque Infection and HSV-2 Detection

**Fig 1:**
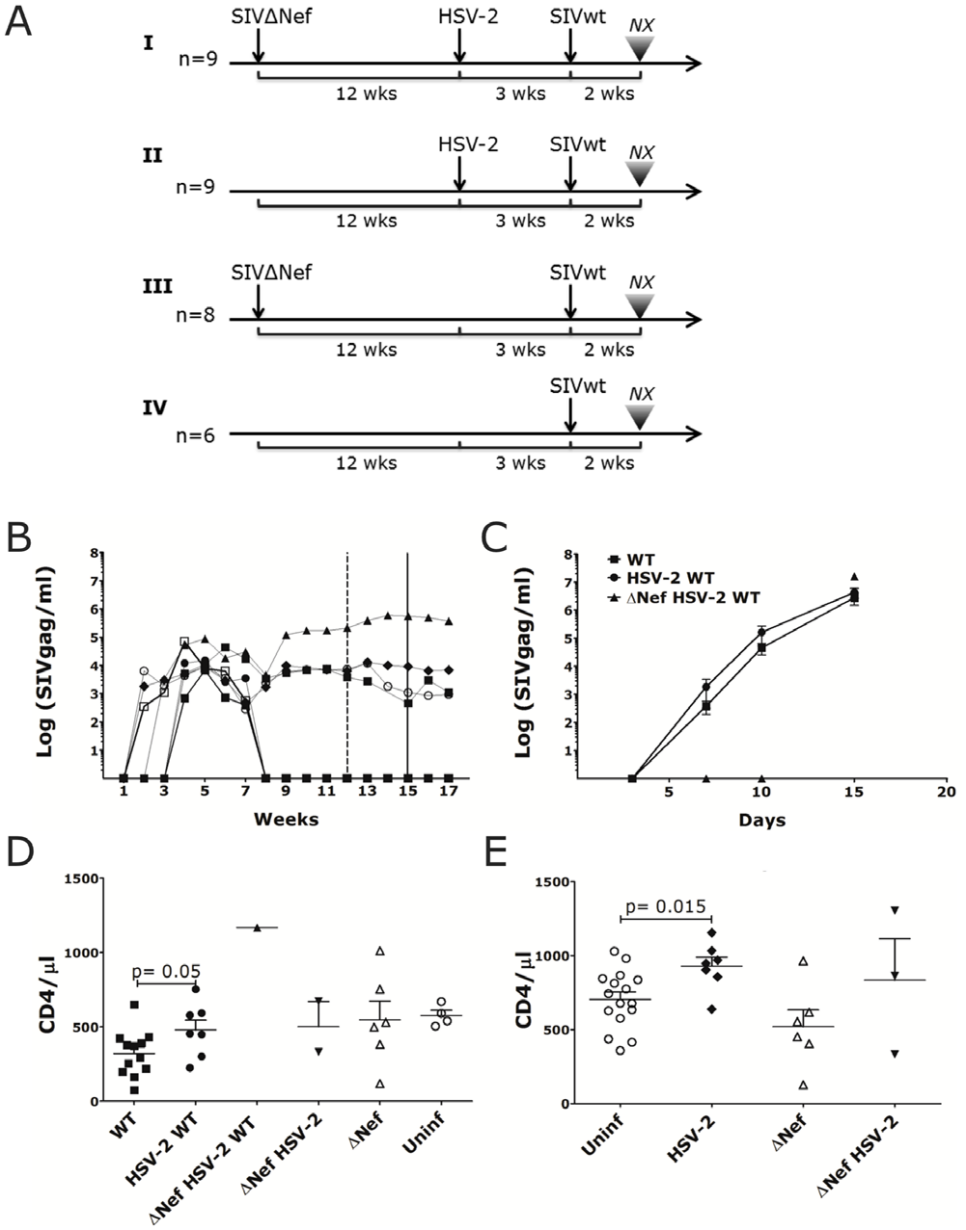
HSV-2/SIVmac239wt co-infected animals have higher acute plasma VL and CD4 counts. A) Schematic representation of the rectal challenges regimen. I. n = 9 animals were inoculated with 3000 TCID_50_ of SIVΔNef, 12 weeks later challenged with 4x10^6^ pfu of HSV-2 and 3 weeks later with 3000 TCID_50_ of SIVmac239wt. II. n = 9 animals were challenged with 4x10^6^ pfu of HSV-2 and 3 weeks later with 3000 TCID_50_ of SIVmac239wt. III) n = 8 animals were inoculated with 3000 TCID_50_ of SIVΔNef and 15 weeks later challenged with 3000 TCID_50_ of SIVmac239wt. IV) 6 animals were challenged with 3000 TCID_50_ of SIVmac239wt as a control. The results of each infection are shown in the S1 Table. B) Plasma SIVgag RNA copies are shown for the SIVΔNef infected animals throughout the study. The dashed and full vertical lines indicate, respectively, the time of HSV-2 and SIVmac239wt challenges. C) Plasma SIVmac239wt (WT) RNA copies detected by discriminatory RT-qPCR are shown post-SIVmac239wt challenge for SIVmac239wt infected animals (WT; mean±SEM n = 12 animals at day 5 and 10, n = 5 at day 15), HSV-2/SIVmac239wt co-infected (HSV-2 WT; mean±SEM 7 animals) and for the 1 SIVΔNef/HSV-2/SIVmac239wt co-infected animal. All animals were necropsied 15 days after SIVmac239wt challenge. D-E) Blood CD4^+^ T cells counts 2 weeks post-WT challenge (D) and 2 weeks post-HSV-2 challenge (E) are shown for the animals that acquired HSV-2 or SIVΔNef infection compared to those that remained uninfected (Uninf). Bars represent mean ± SEM. Significant p values are shown (α<0.05).

The 32 macaques were divided in 4 groups and differentially challenged (See Fig 1A). n = 9 (Group I) were challenged with SIVΔnef (3000 TCID_50_) rectally, 12 weeks later with HSV-2 (4x10^6^pfu) rectally and 3 weeks later with SIVmac239wt (3000 TCID_50_) rectally; n = 9 (Group II) were challenged with HSV-2 and 3 weeks later with SIVmac239wt (both rectally and in parallel with the HSV-2 and SIV challenges of Group I and III); n = 8 (Group III) were challenged rectally with SIVΔnef (3000 TCID_50_) and 15 weeks later with SIVmac239wt (3000 TCID_50_) rectally in parallel with the SIVΔnef and SIVmac239wt challenges of Group I; and n = 6 (Group IV) were challenged rectally with SIVmac239wt only (3000 TCID_50_) in parallel with the other 3 groups as control. The SIVΔnef and SIVmac239wt stocks were prepared in macaque PBMCs and titrated in CEMx174 cells. The generation of HSV-2 strain G stocks was performed on Vero cells (CCL-81; ATCC) [29] and virus titers were determined by plaque-forming assay [29]. For the challenges, viral stocks were diluted in 1mL of PBS and atraumatically inoculated in the rectal cavity. All animals were euthanized at week 17. Rectal swabs were collected as previously described [30]. HSV-2 shedding was detected by nested HSV-PCR on DNA extracted from rectal swabs on days 7, 10 and 14 post HSV-2 challenge, using DNeasy blood and tissue kit (Qiagen, Valencia, CA) following manufacturer's instructions. Six replicates per sample were amplified and a swab sample was considered positive if at least 1 of 6 PCR replicates was positive [12].

### SIV Viral Loads

Plasma samples were obtained from EDTA-treated whole blood and used for the determination of plasma VL by SIV*gag* qRT-PCR[31] until SIVmac239wt challenge. Post SIVmac239wt, Δnef and WT viruses were distinguished by discriminatory qRT-PCR[32] (quantitative Molecular Diagnostics Core, AIDS and Cancer Virus Program Frederick National Laboratory). Tissue viral DNA copy numbers were measured by qPCR by standard curve method. DNA was extracted from LNs and colorectal tissue frozen at the time of necropsy using DNeasy blood and tissue kit (Qiagen) following the manufacturer’s instructions. Primers: SIVgagFW (5’-GGTTGCACCCCCTATGACAT-3’), SIVgagRV (5’-TGCATAGCCGCTTGATGGT-3’); macaque Albumin FW (5’-ATTTTCAGCTTCGCGTCTTTTG-3’) and RV (5’-TTCTCGCTTACTGGCGTTTTCT-3’). Mastermix: ABsolute Blue Q-PCR SYBRGreen low-ROX (Thermo Fisher Scientific, Waltham, MA). The PCR was run on ViiA-7 Real-Time PCR System (Thermo Fisher).

### Detection of Soluble Factors

Immune soluble factors in rectal swabs were determined using the monkey Novex Multiplex Luminex assay (Cytokine Monkey Magnetic 29-Plex Panel; Invitrogen, Waltham, MA) on a MAGPIX^®^ System (Luminex Corporation, Austin, TX). The kit allows the measurement of IL-1RA, GM-CSF, G-CSF, MDC, MIF, I-TAC, FGF-Basic, EGF, HGF, VEGF, Eotaxin, TNFα, IFNγ, IL-1β, IL-2, IL-4, IL-5, IL-6, IL-10, IL-12, IL-15, IL-17, CXCL8, CXCL9, CXCL10, CCL2, CCL3, CCL4 and CCL5. The values of TGF-β1 in plasma were measured by ELISA (R&D Systems, Minneapolis, MN).

### Flow Cytometry

PBMC were isolated from blood by ficoll density centrifugation, while LNs were passed through a 40μm cell strainer. Cell suspensions were stained with the LIVE/DEAD Aqua dye (Molecular probes) before surface and intracellular staining. mAbs included in the DC panel were anti-: CD11c-AF700 (eBioscience, San Diego, CA), CD103-APC (eBioscience), CD123-PCP-Cy5.5, CD80-APCH-7, HLA-DR-BV605 (Invitrogen), CD3-V450, CD14-V450, CD20-V450. α_4_β_7-_PeCy7 and CCR7-AF488 were directly conjugated using Lightning-Link kits (Innova Biosciences, Cambridge, UK). The T cell panel included anti-: CD3-AF700, CD127-APC, CD4-QDot^™^605 (Invitrogen, Waltham MA), PD-1-PCP-efluor710 (eBioscience), α_4_β_7_-PE (clone Act1; NHP Reagent Resource, MassBiologics, University of Massachusetts, Boston, MA), CD95-V450 (all the mAbs were from BD Biosciences, San Jose, CA, unless otherwise indicated). After T cell surface staining, the cells were fixed and permeabilized with fix/perm buffer and incubated with anti-Foxp3-FITC (eBioscience) for 45’ at RT. The regulatory T cells (Treg) population was monitored gating on singlets, live CD3^+^ CD4^+^ FoxP3^+^ CD127^low^ cells although the vast majority of FoxP3^+^ cells were also CD127^low^ and gating only on FoxP3^+^ cells would have given very similar results. Blood T cell responses to SIVmac239 were measured by intracellular staining. Cells were activated on anti- CD49d/CD28 (BD Biosciences) coated plates together with mixes of sequential 15-mer peptides spanning SIVmac239 gag and env (NIH AIDS Reagent Program) or mock solution for 6h. Brefeldin A (Sigma Aldrich, St. Louis, MO) (10μg/ml) was added 1h after the incubation started. Cells were stained for surface markers as described above, fixed and permeabilized using the Fix/Perm Kit (BD Bioscience) and incubated 30’ at RT with a mix of anti-: IL-2, TNFα, IFNγ and IL-17. At least 200,000 events in the live gate were acquired using the BD LSRII Flow Cytometer. Data were analyzed with FlowJo 9.6.1.

### Statistics

The unpaired Mann-Whitney U test (Two-tailed; α = 0.05) was used for comparison of variables between groups of animals. Fisher’s exact test (Two-sided α = 0.05) was used to compare SIVmac239wt acquisition between the groups. Linear regression analysis and the p-value of the Spearman rank coefficient analysis were performed to determine the correlation between various parameters. Statistical analyses were performed using GraphPad Prism vs6 (GraphPad Software).

## Results

### Rectal HSV-2 Infection Increases Rectal SIV Acquisition

In order to understand the impact of rectal HSV-2 infection on the susceptibility to rectal SIVmac239wt and on the ability of SIVΔNef rectal vaccination to protect from SIVmac239wt infection, we devised a four-arm study with 32 macaques (Fig 1A). Group I: 9 animals were inoculated rectally with 3000 TCID_50_ of SIVΔNef, 12 weeks later (after maturation of protective responses[33]) they were challenged with 4x10^8^ pfu of HSV-2 rectally and 3 weeks later, they were challenged rectally with 3000 TCID_50_ of SIVmac239wt; group II: 9 animals were challenged with HSV-2 and 3 weeks later with SIVmac239wt; group III: 8 animals were inoculated with SIVΔNef and 15 weeks later challenged with SIVmac239wt; group IV: 6 animals were challenged rectally with SIVmac239wt in parallel to the other groups as controls. All the animals were euthanized 2 weeks post-SIVmac239wt. The study was performed in 2 rounds, each with 16 animals equally divided among the groups (Round 1 had n = 5 in group I and n = 4 in group II and Round 2 vice-versa). In the 2^nd^ round additional immunologic monitoring was performed by flow cytometry on blood and LN specimens to measure changes in CD4^+^ T cells and dendritic cell (DC) populations. 9 of the 17 animals challenged with SIVΔNef became infected (6 from group I and 3 from group III). 10 of the 18 animals challenged with HSV-2 (5 from group I and 5 from 9 group II) became infected with HSV-2 (HSV-2 shedding could be detected in rectal swabs at one time or more after HSV-2 infection), with SIVΔNef infection not affecting the susceptibility to HSV-2 in this model. The results of the SIVmac239wt infection are summarized in S1 Table.

In Group I, none of the animals that became infected with SIVΔNef, but did not get infected with HSV-2 became infected with SIVmac239wt (0/3), in contrast 1 out of the 3 animals infected with both SIVΔNef and HSV-2 became infected with SIVmac239wt and 2 out of the 2 animals infected with HSV-2-only became infected with SIVmac239wt. The 1 animal that did not acquire SIVΔNef and HSV-2 in group I became infected with SIVmac239wt. In Group II, 5 out of 5 HSV-2 infected animals became infected with SIVmac239wt, in contrast to 1 out of 4 HSV-2 negative animals. In Group III, 3 out of 3 SIVΔNef infected animals were protected against SIVmac239wt, while all 5 animals that did not get infected with SIVΔNef acquired SIVmac239wt. Finally, 5 out of the 6 control macaques in Group IV acquired SIVmac239wt.

In Table 1, the data from all the groups are combined and the results of SIVmac239wt infection are summarized according to the SIVΔNef and HSV-2 status of the animals.

**Table 1:** SIVmac239wt infection status by HSV-2/ SIVΔNef status.

Status	SIVmac239wt+	% Infected
**SIVmac239ΔNef+ HSV-2+**	1/3	33%
**HSV-2+**	7/7	100%
**SIVmac239ΔNef+**	0/6	0%
**Uninfected**	12/16	75%

A total of 32 macaques were challenged rectally with SIVΔNef and HSV-2 (n = 9), with SIVΔNef only (n = 8) or HSV-2 only (n = 9) or were unchallenged (n = 6). 3 Animals became infected with both SIVΔNef and HSV-2, 7 with HSV-2 only and 6 with SIVΔNef only (S1 Table). 16 animals remained uninfected (Uninf group). All animals were challenged rectally with SIVmac239wt and the results of the SIVmac239wt challenge are shown in the table.

100% of the animals infected with HSV-2-only became infected with SIVmac239wt against 75% of SIVmac239wt infected animals in absence of HSV-2 (p = 0.205). In contrast, none of the animals infected only with SIVΔNef became infected with the SIVmac239wt. Thus, in this study, the animals vaccinated/infected with rectal SIVΔNef are fully protected against rectal challenge with SIVmac239wt in the absence of HSV-2 co-infection. In contrast, 1 out of the 3 SIVΔNef/HSV-2 co-infected animals became infected with the SIVmac239wt. The number of animals is too small to generate any conclusion, however the data highlight the need of more studies on the effect of HSV-2 infection on protection conferred by SIVΔNef.

The plasma VL of SIVΔNef infected animals reached values of 10^4^−10^5^ copies/ml (Fig 1B). 2 of the 9 SIVΔNef infected animals (GH44 in the HSV-2/SIVΔNef group I and GI09 in the SIVΔNef-only group III; S1 Table) were already infected with SIVΔNef at the time of SIVΔNef challenge. They were challenged rectally with SIVΔNef ~1 year earlier and, after an initial peak, controlled the virus completely remaining aviremic after the SIVΔNef challenge in this study. 4 out of the 7 macaques that acquired SIVΔNef in this study controlled the virus completely after 8 weeks (Fig 1B). In contrast, the SIVmac239wt plasma VL of SIVmac239wt infected animals reached 10^6^ copies/ml (Fig 1C). Notably, confirming reports of higher acute plasma VL in HSV-2 infected individuals[34], the acute SIVmac239wt plasma VL of the HSV-2 infected macaques was higher, although not significantly, than the VL of HSV-2 uninfected animals (Fig 1C). Plasma viremia was delayed in the SIVΔNef/HSV-2 co-infected animal, but it reached 10^7^ copies/ml at week 2 post-SIVmac239wt. SIV-specific T cell responses could not be detected in blood 10 weeks post-SIVΔNef and 10 days post-SIVmac239wt challenge (probably due to the assay’s poor detection limits). Interestingly, we found that the CD4^+^ T cell blood count was significantly higher in the HSV-2/SIVmac239wt co-infected animals than in the SIVmac239wt–only group (Fig 1D). Since the difference was significant already before SIVmac239wt infection (Fig 1E), but not before HSV-2 challenge (S1 Fig), the data suggest that HSV-2 infection increases the number of blood CD4^+^ T cells and the rate of the initial CD4^+^ T cell decline in HSV-2+/SIVmac239wt+ macaques is similar to that of HSV-2- /SIVmac239wt+. Notably, although we found slightly higher blood VL in the HSV-2/SIVmac239wt co-infected animals, the amount of cell-associated SIVmac239wt in the mesenteric LNs (MLNs) had a tendency to be lower than in the MLNs of HSV-2- animals (p = 0.083) and it was similar in the other LNs and rectal tissue (S2 Fig).

### Rectal HSV-2 Infection Generates a Pro-Inflammatory Environment

**Fig 2:**
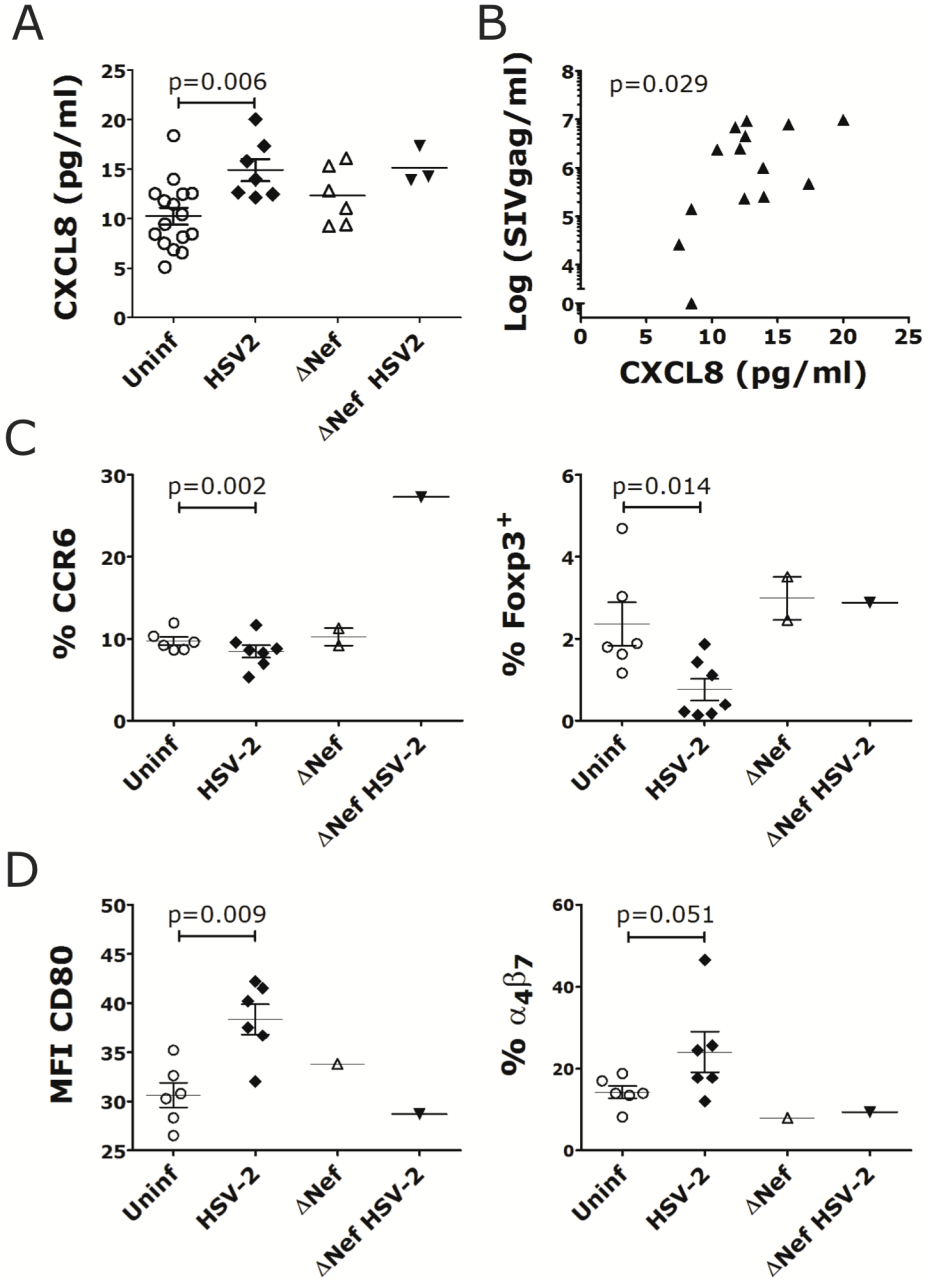
Rectal HSV-2 infection impacts rectal cytokine release and immune cell phenotype in blood and LNs. A) The concentration of CXCL8 in rectal swabs at day 7 post-HSV-2 challenge is shown for the animals that acquired HSV-2 or SIVΔNef infection (or both) compared to those that remained uninfected (Uninf). B) The concentration of CXCL8 in rectal swabs at day 7 post-HSV-2 challenge is plotted against SIV plasma VL at week 2 post-WT challenge for the animals in Round 2 with the exclusion of the SIVΔNef+ ones (plasma VL at week 2 post-WT is missing for Round 1). Spearman correlation p value is shown (significance p<0.05). C) Blood cells were gated on singlets, live and CD3^+^ CD4^+^ cells. The frequency of CCR6^+^ CD4^+^ T cells on day 7 post HSV-2 (left) and FoxP3^+^ CD127^low^ CD4^+^ T cells on day 14 post-HSV-2 (right) is shown for the animals that acquired HSV-2 or SIVΔNef infection compared to those that remained uninfected (Uninf). D) DCs from inguinal LNs were gated on live, singlets, Lin^−^ HLA-DR^+^ cells. The expression of CD80 on total LNs DCs and the frequency of α_4_β_7_^+^ DCs 10 days post-HSV-2 infection are shown for the animals that acquired HSV-2 or SIVΔNef infection compared to those that remained uninfected (Uninf). Bars represent mean±SEM. Significant p values from Mann-Whitney test are shown (α_4_<0.05).

**Fig 3:**
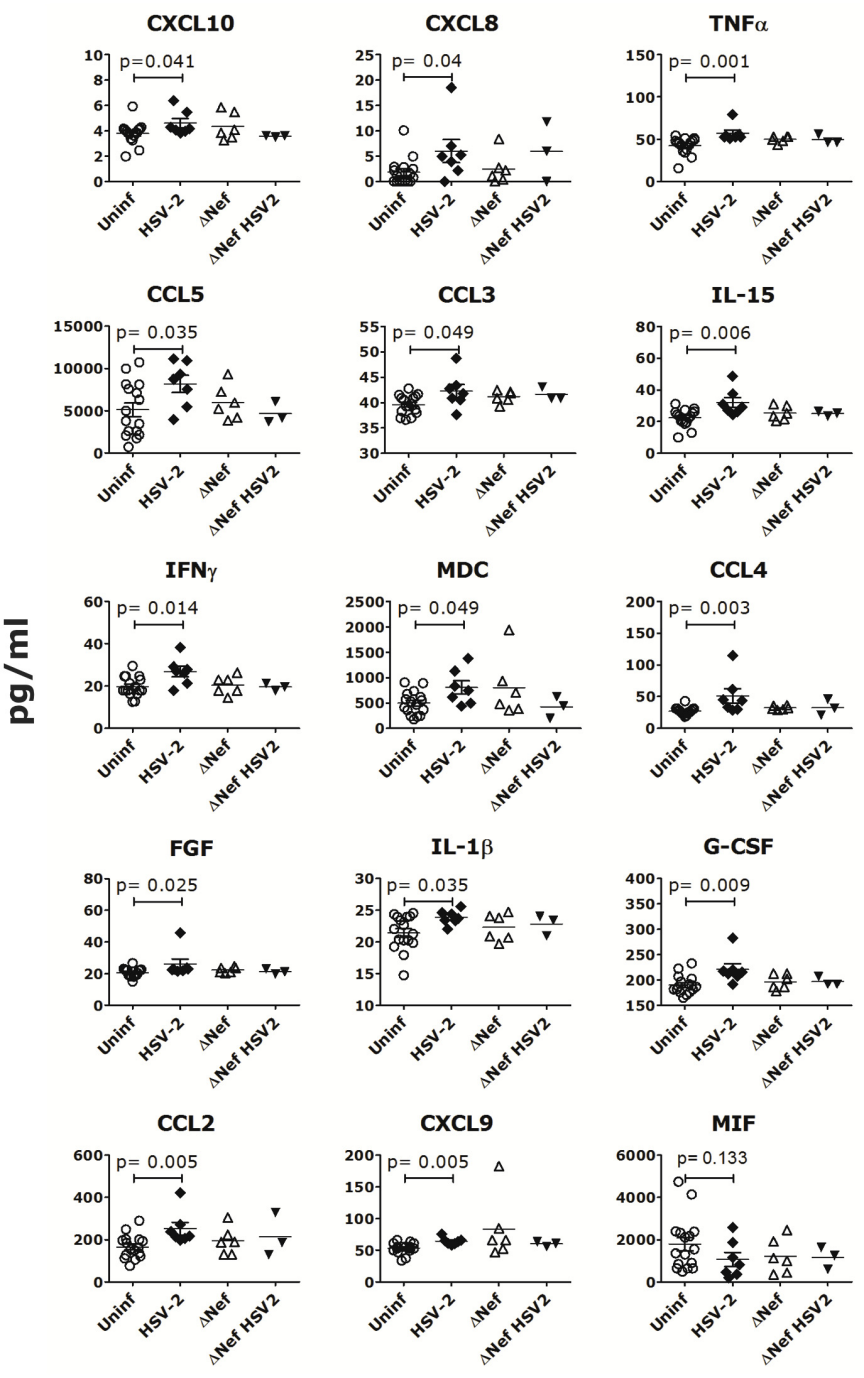
HSV-2 induces a pro-inflammatory environment. The concentration of cytokines and chemokines significantly modulated by HSV-2 infection in plasma 3 weeks post-HSV-2 infection (time of SIVmac239wt challenge) are shown for the animals that acquired HSV-2 or SIVΔNef infection compared to those that remained uninfected (Uninf). Bars represent mean±SEM. Significant p values from Mann-Whitney test are shown (α<0.05).

As seen for vaginal HSV-2 infection[12], rectal HSV-2 infection induced a pro-inflammatory environment locally and systemically. We found significantly higher levels of CXCL8 in the rectal fluids of the HSV-2 infected animals than the uninfected, 7 days post-HSV-2 infection (Fig 2A; other time points were not tested) and there was a similar trend for IL-17, CXCL11 and IL-12 (S3 Fig). Interestingly, the level of CXCL8 in the rectal fluids before SIVmac239wt challenge correlated with the plasma SIVmac239wt VL 2 weeks post-SIVmac239wt infection (Fig 2B) and this was independent of HSV-2 status. Additionally, the frequencies of blood CCR6^+^ CD4^+^ T cells (Fig 2C) and Foxp3^+^ CD127^low^ CD4^+^ T cells (Fig 2C and gating strategy in S4 Fig) were lower in the HSV-2 infected animals compared to uninfected respectively 7 and 14 days post-HSV-2 infection. Moreover, the DCs in the inguinal LNs of HSV-2 infected animals expressed higher CD80 and contained more α_4_β_7_^+^ DCs 10 days after HSV-2 infection (Fig 2D). Finally, at the time of SIVmac239wt challenge (3 weeks post-HSV-2), the HSV-2 infected animals had significantly higher levels of certain inflammatory cytokines in plasma than uninfected animals (Fig 3). Interestingly, among the 3 animals co-infected with HSV-2 and SIVΔNef, the one animal that acquired SIVmac239wt infection was also the one with the highest levels of all the soluble factors shown in Fig 3. The only factor that showed an inverse tendency (lower levels in the HSV-2 infected animals) was the macrophage migration inhibitory factor, MIF (Fig 3).

### HSV-2/SIVmac239wt Co-Infected Macaques Have a Higher Pro-Inflammatory Environment

**Fig 4:**
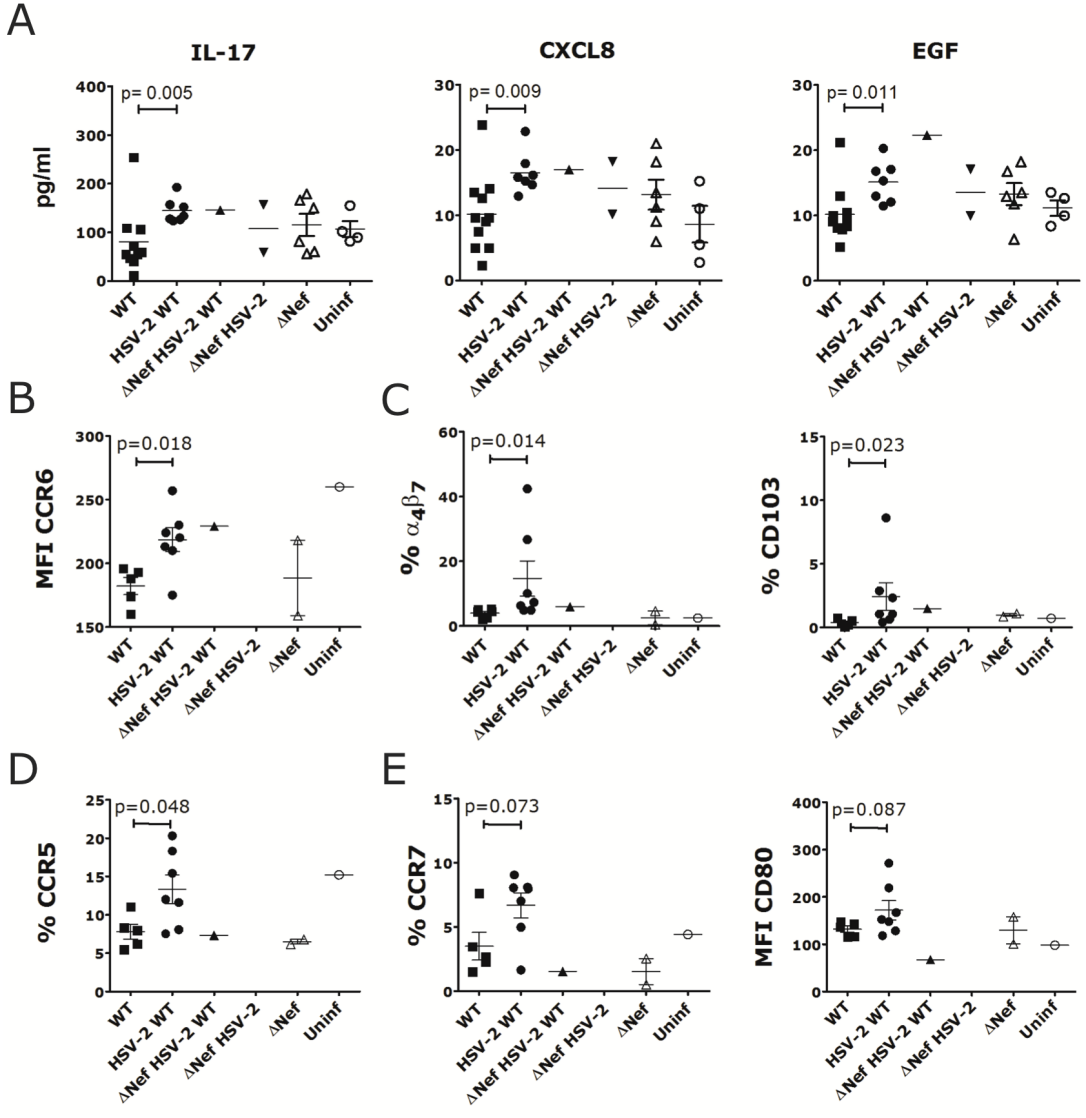
HSV-2/SIV co-infected macaques have a higher pro-inflammatory environment. A) The levels of cytokines and chemokines significantly modulated by HSV-2 co-infection in rectal swabs on day 7 post-SIVmac239wt challenge are shown for all animals in round 1 and 2 that acquired HSV-2, SIVΔNef or SIVmac239wt infection (all 3 or any combination of them) compared to those that remained uninfected (Uninf). B-C) Cells from iliac LNs were gated on live, singlets and CD3^+^ CD4^+^ (B) or Lin^-^ HLA-DR^+^ (C) cells. The expression of CCR6 on CD4^+^ T cells and the frequencies of α_4_β_7_^+^ DCs and CD103^+^ DCs are shown for all animals in round 2 that acquired HSV-2, SIVΔNef and/or SIVmac239wt infection compared to the one that remained uninfected (Uninf) D-E) Cells from MLNs were gated on live, singlets and CD3^+^ CD4^+^ (D) or Lin^-^ HLA-DR^+^ (E) and the frequency of CCR5^+^ cells within CD95^+^ CD4^+^ T cells (D) and CCR7^+^ DCs and the expression of CD80 on DCs (E) are shown for all animals in round 2 that acquired HSV-2, SIVΔNef and/or SIVmac239wt infection compared to the one that remained uninfected (Uninf). Bars represent mean±SEM. Significant p values from Mann-Whitney test are shown (α<0.05). p<0.125 are also shown to indicate a tendency toward a significant difference.

Next, we investigated if together with driving higher acute plasma VL, HSV-2 infection had other effects on SIVmac239wt infection. HSV-2/SIVmac239wt co-infected animals had significantly higher levels of IL-17, CXCL8 and epidermal growth factor (EGF) in their rectal swab than the SIV-only animals, 7 days post-SIVmac239wt infection (Fig 4A; plasma not available), while no significant difference was found in plasma 2 weeks post-SIV infection (swabs not available). No differences were noted in the levels of TGF-β between HSV-2 infected and uninfected after SIVmac239wt challenge (S5 Fig). Moreover, while there were no differences in all major CD4^+^ T cell and DC subsets in blood, in the iliac LNs of the HSV-2/SIVmac239wt co-infected animals the CD4^+^ T cells expressed higher levels of CCR6 (Fig 4B) and there were higher frequencies of α_4_β_7_^+^ and CD103^+^ DC subsets (Fig 4C). Interestingly, paralleling a tendency toward lower cell associated VL, the MLN of the co-infected animals retained more CCR5^+^ CD4^+^ T cells (Fig 4D). Indeed, there was a significantly higher frequency of CCR5^+^ memory CD4^+^ T cells in the MLN of the HSV-2/SIV co-infected animals, more similar to the level of the uninfected than in the MLN of the SIVmac239wt-only infected animals (Fig 4D). Finally, the frequency of CCR7^+^ DCs and CD80 levels on DCs in the MLN of the co-infected animals tended to be higher (p = 0.087; Fig 4E).

## Discussion

We have previously shown that HSV-2 vaginally infected macaques are more susceptible to SHIVSF162P3 infection in vivo and this correlates with an HSV-2-driven increase in the expression of α_4_β_7_ on CD4^+^ T cells ex vivo[12]. In the present study, we focused on rectal HSV-2 infection, determining if it would increase susceptibility to rectal SIV infection even after the use of a benchmark SIV vaccine, the LAV SIVΔNef. We also obtained the first evidence for the protective effect of rectal SIVΔNef vaccination against the homologous SIVmac239wt rectal challenge. Intravenous SIVΔNef inoculation is 100% effective against intravenous and mucosal challenge with SIVmac239[35, 36]. In contrast, SIVΔNef vaccination via tonsillar route confers only partial protection against rectal SIVmac251[37, 38]. In our study, rectal vaccination with SIVΔNef completely protected HSV-2 negative animals from SIVmac239wt acquisition via rectal route. This is in contrast to one out of three animals co-infected with SIVΔNef and HSV-2, which acquired SIVmac239wt infection. Although this SIVΔNef+/HSV-2+ animal was among the SIVΔNef controllers, also the other 2 HSV-2/SIVΔNef co-infected macaques and an additional 3 SIVΔNef-only infected animals were aviremic at the time of SIVmac239wt challenge. Instead, the SIVΔNef+/HSV-2+ animal that acquired SIVmac239wt had higher levels of pro-inflammatory cytokines compared to the other SIVΔNef/HSV-2 co-infected animals, suggesting a role of inflammation in decreasing SIVΔNef protection. A higher pro-inflammatory environment is also associated with the increased susceptibility of all the SIVΔNef negative HSV-2 infected animals. Indeed, elevated inflammatory cytokines at mucosal surfaces have been associated with HIV susceptibility and with increased frequency of endocervical CD4^+^ T cells [39–41]. The importance of the level of inflammation is highlighted by the strong correlation between the levels of CXCL8 in rectal fluids 2 weeks before SIVmac239wt challenge and the plasma VLs 2 weeks post-SIVmac239wt infection. Thus, it appears that higher inflammatory state may, not only increases susceptibility to SIV, but it may drive viral replication at least systemically. This is important, since higher inflammatory state possibly contributes to the increased prevalence of HIV infection in Sub-Saharan Africa[41]. Finally, the HSV-2-driven decrease in the frequency of blood Treg and the increased expression of CD80 and α_4_β_7_ on DCs in the inguinal LNs also suggest that one of the main effects of HSV-2 rectal infection is the induction of a generalized pro-inflammatory state. The small decrease in the frequency of blood CCR6 may be due to a redistribution of these cells in the tissue at the site of HSV-2 infection. Since, CCR6^+^ CD4^+^ T cells are highly susceptible to HIV/SIV infection[42], this would agree with the findings that HSV-2 increased the frequency of susceptible cells at the mucosal site of challenge[13, 14]. Unfortunately, rectal samples post-HSV-2 infection were not collected and this hypothesis as well as the HSV-2-driven increase in α_4_β_7_^+^ CD4^+^ T cells at the rectal site of infection, which we previously described[14], could not be verified. Interestingly, in contrast to the higher plasma VL, we found that the MLNs of the HSV-2/SIVmac239wt co-infected animals had non-significantly lower cell-associated SIVmac239wt than HSV-2 uninfected animals. This agrees with a less pronounced depletion of CCR5^+^ CD4^+^ T cells in this site. Thus, it appears that HSV-2 co-infection drives SIVmac239wt replication in blood, but not in the LNs. Moreover, we found that the HSV-2/SIVmac239wt co-infected animals had higher concentration of pro-inflammatory factors in the rectal fluids than the SIVmac239wt-only animals. However, this was at a time post-SIVmac239wt infection when the SIVmac239wt-only infected animals did not have higher concentration of pro-inflammatory factors than uninfected animals. It is possible, that later on SIVmac239wt would drive an increase in inflammatory factor, decreasing or ablating the difference between the SIVmac239wt single infected and the HSV-2/SIVmac239wt co-infected animals. Our results from the vaginal ex vivo studies in [12] indeed suggest that HSV-2 co-infection may suppress the release some of the SIV-driven increased inflammatory factors (S2 Table in [12]). This needs to be confirmed in studies with a longer follow-up after SIV infection. Interestingly, HSV-2 co-infection had no significant impact on the phenotype of immune cells in blood in SIV infected animals, but it had some effect at the level of iliac and MLNs. This may translate in differences in the immune response to SIV in the HSV-2/SIV co-infected animals and should also be investigated in studies with longer follow-up. T cells systemic responses to SIVΔNef were undetectable and, in any case, with only 3 animals co-infected with SIVΔNef and HSV-2, it would not have been possible to determine if HSV-2 had an impact on T cell responses to SIVΔNef. Since LNs T cell responses have been linked with SIVΔNef protection after i.v. infection[36], future studies will need to address if rectal SIVΔNef induces stronger T cell responses in the LNs than in blood, and if they correlate with protection, addressing the effect of HSV-2 co-infection.

In conclusion, our results suggest that rectal HSV-2 infection may increase susceptibility to rectal SIV infection generating a pro-inflammatory environment and increasing systemic SIV replication during the acute phase. Moreover, our data support the need of investigating the impact of HSV-2 infection on the protective effect of HIV vaccines and on HIV-specific responses. Studies with larger number of animals are needed to confirm our results on the effect of HSV-2 rectal infection on susceptibility to SIV and on its effect on viral and immunological parameters in chronic SIV infection.

## Supporting Information

S1 Fig CD4 counts at the time of HSV-2 challenge. CD4^+^ T cell numbers in blood before HSV-2 challenge (after SIVΔNef) challenge for all animals according to their SIVΔNef status and their future HSV-2 status.
(PDF)

S2 Fig Tissue SIV Loads. Copies of cell-associated SIVgag DNA in tissue is shown for all animals that acquired HSV-2, SIVΔNef and/or SIVmac239wt infection compared to the one that remained uninfected (Uninf) at necropsy. Bars represent mean±SEM.
(PDF)

S3 Fig Pro-inflammatory environment in rectal tissue of HSV-2 infected animals. The concentration of cytokines and chemokines that had a tendency to differ between HSV-2 infected and HSV-2 uninfected in rectal swabs 7 days post HSV-2 challenge are shown for all animals that acquired HSV-2 and/or SIVΔNef infection compared to the one that remained uninfected (Uninf). Bars represent mean±SEM. p<0.125 are shown to indicate a tendency toward a significant difference (p<0.05 is considered significant).
(PDF)

S4 Fig Treg gating strategy. Singlets, live, CD3^+^ CD4^+^ T cells were gated on Foxp3^+^ CD127^low^ cells.
(PDF)

S5 Fig Plasma TGF-β1 7 days post-SIVwt challenge. Plasma aliquots frozen right after collection were thawed and the concentration of total TGF-β1 was measured by ELISA (R&D) following manufacturer’s instruction. The concentration of total TGF-β1 in plasma of all animals that acquired HSV-2, SIVΔNef and/or SIVmac239wt infection compared to the one that remained uninfected (Uninf) is shown. Bars represent mean±SEM.
(PDF)

S1 Table List of all the macaques in the study with challenges and infection status. 32 Indian rhesus macaques were divided into 4 groups and challenged (Group I and III only) with SIV_mac239_ΔNef rectally, 12 weeks later they were challenged (Group I and II only) with HSV-2 (4x10^6^pfu) rectally and 3 weeks later all animals were challenged with SIVmac239wt (3000 TCID_50_) rectally. Each monkey’s final infection status is reported for each virus (based on plasma VL for SIV and at least 1 positivity to HSV-2 nested PCR in rectal swabs for HSV-2).
(DOCX)
