# Supplementary figures and images for: Rectal HSV-2 Infection May Increase Rectal SIV Acquisition Even in the Context of SIVΔnef Vaccination
Source: PLoS One. 2016 Feb 17;11(2):e0149491. doi: 10.1371/journal.pone.0149491 (PMC4757571; doi:10.1371/journal.pone.0149491)

S1 Fig

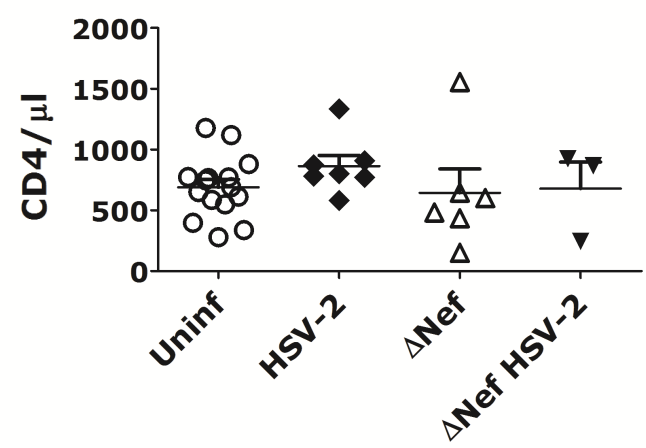

Supplement: S1 Fig — CD4+ T cell numbers in blood before HSV-2 challenge (after SIVΔNef) challenge for all animals according to their SIVΔNef status and their future HSV-2 status. (PDF) [file pone.0149491.s001.pdf]

S2 Fig

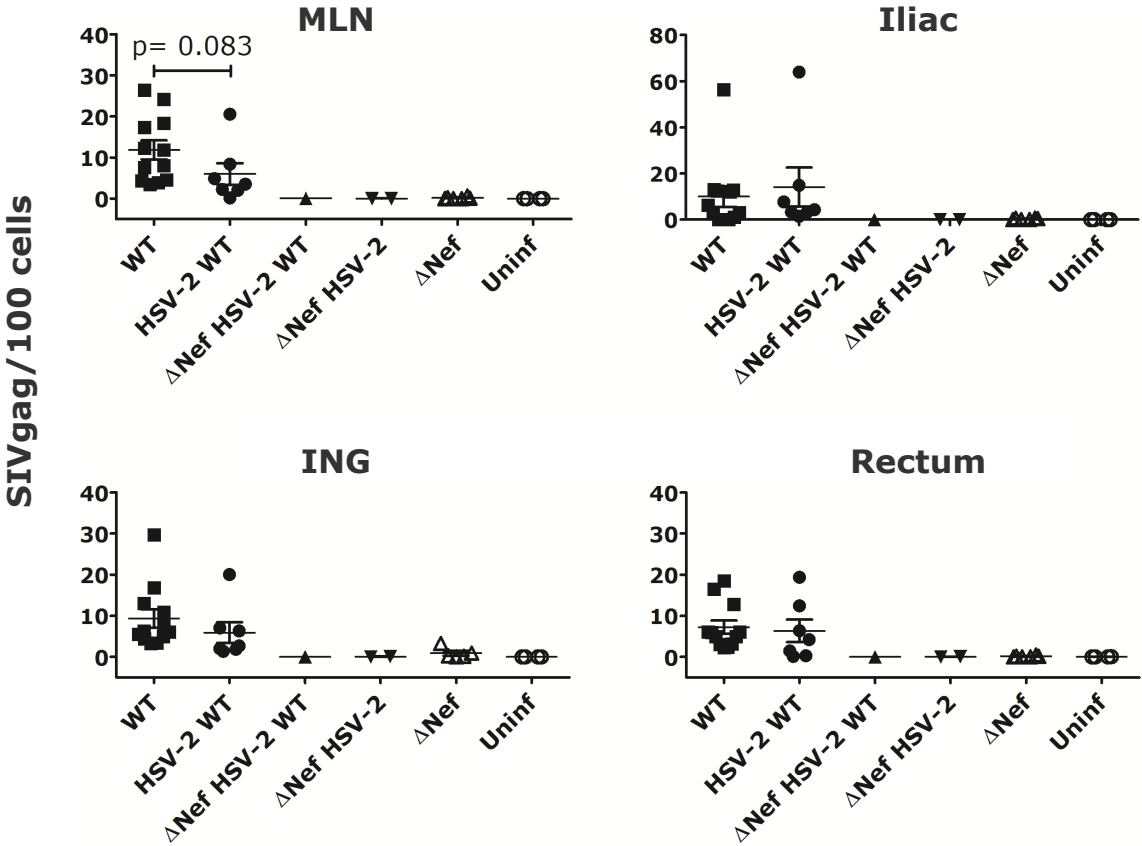

Supplement: S2 Fig — Copies of cell-associated SIVgag DNA in tissue is shown for all animals that acquired HSV-2, SIVΔNef and/or SIVmac239wt infection compared to the one that remained uninfected (Uninf) at necropsy. Bars represent mean±SEM. (PDF) [file pone.0149491.s002.pdf]

S3 Fig

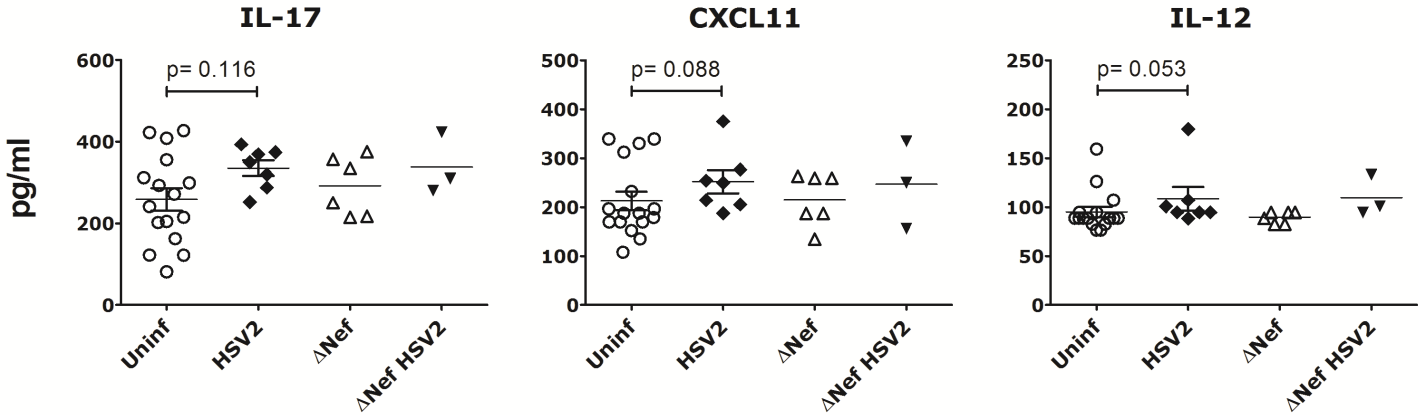

Supplement: S3 Fig — The concentration of cytokines and chemokines that had a tendency to differ between HSV-2 infected and HSV-2 uninfected in rectal swabs 7 days post HSV-2 challenge are shown for all animals that acquired HSV-2 and/or SIVΔNef infection compared to the one that remained uninfected (Uninf). Bars represent mean±SEM. p<0.125 are shown to indicate a tendency toward a significant difference (p<0.05 is considered significant). (PDF) [file pone.0149491.s003.pdf]

S4 Fig

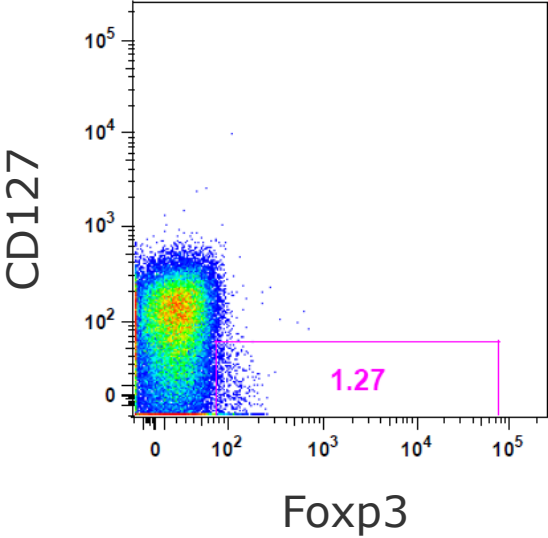

Supplement: S4 Fig — Singlets, live, CD3+ CD4+ T cells were gated on Foxp3+ CD127low cells. (PDF) [file pone.0149491.s004.pdf]

S5 Fig

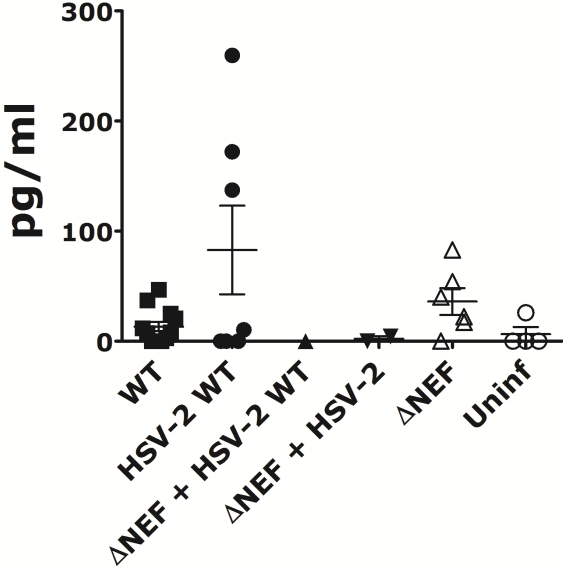

Supplement: S5 Fig — Plasma aliquots frozen right after collection were thawed and the concentration of total TGF-β1 was measured by ELISA (R&D) following manufacturer’s instruction. The concentration of total TGF-β1 in plasma of all animals that acquired HSV-2, SIVΔNef and/or SIVmac239wt infection compared to the one that remained uninfected (Uninf) is shown. Bars represent mean±SEM. (PDF) [file pone.0149491.s005.pdf]
