# Supplementary material for: Rectal HSV-2 Infection May Increase Rectal SIV Acquisition Even in the Context of SIVΔnef Vaccination
Source: PLoS One. 2016 Feb 17;11(2):e0149491. doi: 10.1371/journal.pone.0149491 (PMC4757571; doi:10.1371/journal.pone.0149491)
Supplement: S1 Table — 32 Indian rhesus macaques were divided into 4 groups and challenged (Group I and III only) with SIVmac239ΔNef rectally, 12 weeks later they were challenged (Group I and II only) with HSV-2 (4x10^6^pfu) rectally and 3 weeks later all animals were challenged with SIVmac239wt (3000 TCID50) rectally. Each monkey’s final infection status is reported for each virus (based on plasma VL for SIV and at least 1 positivity to HSV-2 nested PCR in rectal swabs for HSV-2). (DOCX) [file pone.0149491.s006.docx]

**S1 Table.**

| Monkey | Challenge | | | Group | SIVΔNef | HSV-2 | SIV_mac239wt_ |
| --- | --- | --- | --- | --- | --- | --- | --- |
| GL35 | SIV_mac239 ΔNef_ | HSV-2 | SIV_mac239 (WT)_ | I | - | - | + |
| EN81 | SIV_mac239 ΔNef_ | HSV-2 | SIV_mac239 (WT)_ |  | + | - | - |
| DT23 | SIV_mac239 ΔNef_ | HSV-2 | SIV_mac239 (WT)_ |  | + | + | - |
| GI67 | SIV_mac239 ΔNef_ | HSV-2 | SIV_mac239 (WT)_ |  | + | - | - |
| *GH44 | SIV_mac239 ΔNef_ | HSV-2 | SIV_mac239 (WT)_ |  | + | + | - |
| EB34 | SIV_mac239 ΔNef_ | HSV-2 | SIV_mac239 (WT)_ |  | - | + | + |
| GK95 | SIV_mac239 ΔNef_ | HSV-2 | SIV_mac239 (WT)_ |  | + | + | + |
| CL23 | SIV_mac239 ΔNef_ | HSV-2 | SIV_mac239 (WT)_ |  | + | - | - |
| FF90 | SIV_mac239 ΔNef_ | HSV-2 | SIV_mac239 (WT)_ |  | - | + | + |
| DV36 |  | HSV-2 | SIV_mac239 (WT)_ | II | na | - | - |
| GI97 |  | HSV-2 | SIV_mac239 (WT)_ |  | na | - | + |
| FH32 |  | HSV-2 | SIV_mac239 (WT)_ |  | na | - | - |
| EK92 |  | HSV-2 | SIV_mac239 (WT)_ |  | na | - | - |
| FE39 |  | HSV-2 | SIV_mac239 (WT)_ |  | na | + | + |
| EE60 |  | HSV-2 | SIV_mac239 (WT)_ |  | na | + | + |
| EA71 |  | HSV-2 | SIV_mac239 (WT)_ |  | na | + | + |
| GR50 |  | HSV-2 | SIV_mac239 (WT)_ |  | na | + | + |
| HH82 |  | HSV-2 | SIV_mac239 (WT)_ |  | na | + | + |
| FE87 | SIV_mac239 ΔNef_ |  | SIV_mac239 (WT)_ | III | - | na | + |
| EE34 | SIV_mac239 ΔNef_ |  | SIV_mac239 (WT)_ |  | - | na | + |
| GM84 | SIV_mac239 ΔNef_ |  | SIV_mac239 (WT)_ |  | + | na | - |
| *GI09 | SIV_mac239 ΔNef_ |  | SIV_mac239 (WT)_ |  | + | na | - |
| GJ12 | SIV_mac239 ΔNef_ |  | SIV_mac239 (WT)_ |  | - | na | + |
| FG44 | SIV_mac239 ΔNef_ |  | SIV_mac239 (WT)_ |  | + | na | - |
| GN96 | SIV_mac239 ΔNef_ |  | SIV_mac239 (WT)_ |  | - | na | + |
| GK53 | SIV_mac239 ΔNef_ |  | SIV_mac239 (WT)_ |  | - | na | + |
| EN40 |  |  | SIV_mac239 (WT)_ | IV | na | na | + |
| CP50 |  |  | SIV_mac239 (WT)_ |  | na | na | + |
| CP74 |  |  | SIV_mac239 (WT)_ |  | na | na | + |
| HI31 |  |  | SIV_mac239 (WT)_ |  | na | na | + |
| FC56 |  |  | SIV_mac239 (WT)_ |  | na | na | - |
| FD01 |  |  | SIV_mac239 (WT)_ |  | na | na | + |

***** Already SIVΔNef infected at the time of SIVΔNef challenge
